# The Biology of Exosomes in Breast Cancer Progression: Dissemination, Immune Evasion and Metastatic Colonization

**DOI:** 10.3390/cancers12082179

**Published:** 2020-08-05

**Authors:** Cinzia Giordano, Giusi La Camera, Luca Gelsomino, Ines Barone, Daniela Bonofiglio, Sebastiano Andò, Stefania Catalano

**Affiliations:** 1 Department of Pharmacy, Health and Nutritional Sciences, Via P Bucci, University of Calabria, 87036 Arcavacata di Rende (CS), Italy; giusylacamera93@gmail.com (G.L.C.); luca.gelsomino@unical.it (L.G.); ines.barone@unical.it (I.B.); daniela.bonofiglio@unical.it (D.B.); sebastiano.ando@unical.it (S.A.); 2 Centro Sanitario, Via P Bucci, University of Calabria, 87036 Arcavacata di Rende (CS), Italy

**Keywords:** exosomes, extracellular vesicles, breast cancer, metastasis, EMT

## Abstract

In recent decades, the study of exosome biology has gained growing interest, representing an active area of cancer research with many potential clinical applications. Exosomes are small lipid bilayer particles released by cells with pleiotropic functions that have been reported to regulate the complex intracellular pathway involved in all steps of breast cancer development—from initiation to progression toward a metastatic dissemination. Particularly, the role of these microvesicles has been explored in metastasis, which represents the leading cause of breast cancer morbidity and mortality worldwide. Reports highlight that the plasticity of breast cancer cells, fundamental for the establishment of distant metastasis, may be in part attributed to exosome-carried signals shared between adjacent cells and long-distance cells in the body. In the present review, we will discuss the functions of exosomes in the metastatic breast cancer process and secondary site outgrowth. The possibility to decode the exosome functions in advanced diseases may offer new opportunities for early detection, molecular targeted therapies and exosome-based therapeutics in breast cancer.

## 1. Introduction

Female breast cancer accounts for 11.6% of the total cancer incidence burden and for 6.6% of the cancer-related deaths burden worldwide. It is the most common cancer among women, with more than 2 million (24.2%) newly diagnosed cases in both developed and developing countries, and represents the leading cause of cancer mortality; more than 0.6 million (15.0%) women died in 2018 as reported in the GLOBOCAN database [1]. Despite an increased rate of early detection of the disease and the improvement in the clinical management of patients, the number of women who die from this disease still remains too high due to treatment failure and the development of deadly metastatic disease [2,3]. Indeed, between 20% and 30% of women with breast cancer will develop metastatic disease. Depending on the molecular subtype, the treatment options for metastatic breast cancer are largely focused on: endocrine therapy for patients with human epidermal growth factor receptor 2 (HER2)-negative luminal metastatic tumors; anti-HER2 therapy for patients with HER2-positive metastatic tumors; systemic chemotherapies, in triple negative breast cancer or in highly symptomatic patients [4]. Thus, the main challenge in breast cancer treatment is to control the establishment of metastasis at secondary sites, mainly responsible for patient mortality. This is the reason why a large amount of research is currently focused on improving the knowledge on the biological and molecular mechanisms underlying the metastatic processes in breast cancer.

A large body of evidence, in recent decades, has highlighted that cell-to-cell communication represents a key driver of cellular functions and tissue homeostasis in both physiological and pathological conditions, including cancer. As important and well-established mediators of intercellular communication, the membrane-derived vesicles and, particularly, exosomes [5], have obtained growing attention since they appeared as essential players in the initiation, progression and metastatic processes in breast cancer [6,7,8]. Several findings have shown that exosomes, able to communicate both at a short- and long-distance, by releasing their genetic and molecular cargo to the recipient cells, profoundly influence many aspects of the metastatic cascade. 

Here, we will summarize and critically discuss the current advancements in understanding the role of exosomes in the metastatic cascade of breast cancer. Particularly, we will highlight both homotypic- (cancer cell-to-cancer cell) and heterotypic-transfer (cancer cell-to-stromal cells and vice versa) of bioactive molecules and signalings able to modulate the key steps of the metastatic process. Because of the difficulty of assigning the correct nomenclature to the lipid bilayer particles released by cells in the different experimental conditions, as claimed by the “International Society of Extracellular Vesicles” [9], some scientific publications referred to “Extracellular Vesicles (EVs)” as a collective term covering small extracellular vesicles including exosomes. Thus, in the present review, findings related to EVs in the context of metastatic breast cancer were also reported.

## 2. Characteristics of Exosomes

### 2.1. Exosome Structure and Contents

Exosomes are endocytic membrane vesicles of 30–150 nm in size that are released by various cell types into extracellular spaces, including body fluids such as blood, lymph, cerebrospinal fluid, urine and saliva. These extracellular vesicles are functional vehicles that carry a complex cargo of nucleic acids, proteins and enzymes, depending on the nature and status of original cells. 

A large cargo of nucleic acids, that can be incorporated into recipient cells, has been identified in exosomes. MicroRNAs (miRNA) and mRNAs are the most important cargos packed into exosomes, but these vesicles also contain a considerable amount of RNAs of other types, such as transfer RNA, ribosomal RNA and small nuclear RNA, small nucleolar RNA, piwi-interacting RNA and long non-coding RNA. MiRNAs and mRNAs packed into exosomes shuttled from donor to recipient cells, actively modulate the activities of recipient cells inducing the post-transcriptional regulation of gene expression and transient or persistent phenotypic change [10]. 

**Figure 1:**
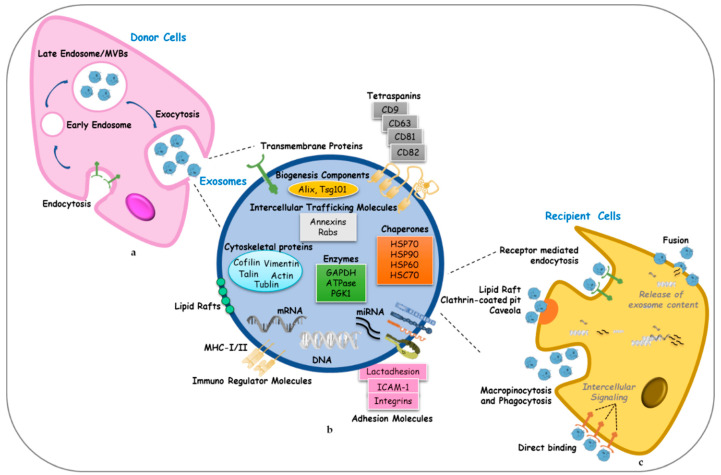
Biogenesis, structure and uptake of exosomes. (**a**) Exosome biogenesis starts with membrane endocytosis resulting in the formation of early endosomes that subsequently mature into late endosomes or Multi Vesicular Bodies (MVBs). MVBs can be transported to the plasma membrane through the cytoskeletal network and exocytosis occurs, allowing the release of exosomes into the extracellular space. (**b**) Exosomes contain different families of proteins categorized according to their functions, lipids, metabolites and nucleic acids (DNA, RNA, miRNA and other non-coding RNAs). (**c**) The entry of exosomes in recipient cells can be mediated by several mechanisms: direct fusion of exosomes with the plasma membrane allowing the deposit of their content into the cytoplasm; receptor-mediated endocytosis, lipid rafts, clathrin-coated pit and caveolae, macropinicytosis, phagocytosis, that allow the entry of intact exosomes in the cells; direct binding of exosomes to specific receptors that induce intracellular signalings into recipient cells. HSC: heat shock cognate; HSP: heat shock protein; ICAM-1: intercellular adhesion molecule 1; MHC: major histocompatibility complex; PGK1: phosphoglycerate kinase 1; Rab: Ras-related proteins in brain; Tsg101: Tumor susceptibility gene 101.

Different proteins were conserved in exosomes, regardless of their cellular origin [11]. Indeed, the exosomal membrane surface has been verified to contain proteins required for their transport and binding to target cells as GTPases and annexins. The lipid bilayer membrane structures of exosomes also carry typical transmembrane proteins and receptors, such as the transmembrane proteins PGRL, lysosome associated membrane protein-1/2 (LAMP1/2), CD13; adhesion molecules such as intercellular adhesion molecule 1 (ICAM-1), Lactadhesion and integrin; the lipid raft associated proteins such as Flottilin or Cholesterol; and tetraspanins, most characteristically CD63 and CD81, which are found in the majority of the exosomes regardless of their origin (Figure 1). In addition, an exosome’s surface exhibits immunoregulator molecules, including major histocompatibility complex (MHC)-I and -II, CD80 and CD86 that are cell-type-specific proteins [11,12].

Exosomes are enriched in lipids that play a vital role in their biogenesis and are characteristic of the cell origin. Generally, phosphatidylserine (PS), phosphatidic acid, cholesterol, sphingomyelin (SM), arachidonic acid and other fatty acids, prostaglandins and leukotrienes, ensure the stability and structural rigidity of the exosomes. Interestingly, lipids such as lyosbisphosphatidic acid (LBPA) are found in the internal membranes of multivesicular bodies (MVBs) and play an important role in the biogenesis of exosomes [13]. 

### 2.2. Exosome Biogenesis

Exosome biogenesis begins within the endosomal system, where early endosomes mature into late endosomes or MVBs, and the inward budding of the endosomal membranes produces an accumulation of intraluminal vesicles (ILVs) in the lumen of the organelles. During this process, transmembrane proteins are integrated into the invaginating membrane, while the cytosolic components are enclosed within the ILVs. The endosomal sorting complex required for transport (ESCRT) is a protein machinery composed of successive complexes (ESCRT-0, -I, -II and -III) associated with the membrane of MVBs. All components work cooperatively to regulate the formation of vesicle budding and protein cargo sorting in a ubiquitinylation-dependent manner [14,15]. The ESCRT mechanism starts by ESCRT-0 that recognizes and sequestrates ubiquitinated proteins in the late endosomal membrane. After the initial involution of the limiting membrane into the MVB lumen triggered by ESCRT-I/II, ESCRT-III promotes the budding process and cleaves the buds to form ILVs. Hence, the ATPase Vps4 drives the splitting of the ESCRT-III complex from the MVB membrane. Sorting of proteins into ILVs can also occur independently of ubiquitination. Recent studies showed a new mechanism that involves syntenin and syndecans [16]. In addition, different evidence suggests an ESCRT-independent mechanism for exosome biogenesis that involves exosomal lipids [17]. After maturation, MVBs can fuse with the plasma membrane and release the enclosed ILVs, which are referred to as “exosomes”, into the extracellular space. Alternatively, these vesicles are trafficked to lysosomes and their cargo is degraded [18]. 

The mechanisms of exosome biogenesis appear deregulated in cancer, resulting in an increased amount of vesicles released by cancer cell lines as well as recovered in the blood of patients with cancer [19,20]. Particularly, in breast cancer, it has been demonstrated that the release of exosomes from the human tumor cell line B42 clone 16 was significantly higher compared to the amount of exosome released by the parental normal mammary epithelial cells—HMEC B42 [21]. In addition, in vitro studies underlined an increased exosome release in cells maintained in cellular stress conditions such as hypoxia [22], and revealed that exosome secretion can be modulated by the Ca^2+^/Ca^2+^-dependent Rab binding protein (Munc13-4) pathway [23]. More recently, we demonstrated that the adipokine leptin may represent an additional important inducer of exosome release in breast cancer. Indeed, we have found that the leptin/leptin receptor axis, modulating the expression of the leptin target gene heat shock protein 90, was able to induce the protein expression of the tumor susceptibility gene 101 (Tsg101), a key component of the ESCRT-I complex. This mechanism results in an increase in MVB formation and exosome secretion in both estrogen receptor positive MCF-7 and triple negative MDA-MB-231 breast cancer cells [24]. 

### 2.3. Exosome Uptake

The molecular mechanisms involved in exosome internalization into recipient cells are still unclear. Exosome internalization might occur by several biological mechanisms comprising fusion with the plasma membrane, receptor-mediated endocytosis, macropinocytosis and phagocytosis. The first step of exosome uptake involves interaction with the recipient cell near or distant to the donor cell. It is still not completely understood whether this process depends on a specific combination of EV subtype and acceptor cell. The second step of uptake is the internalization into the cell that could occur through a non-specific process such as macropinocytosis or micropinocytosis, or through a specific receptor-dependent pathway [25]. Exosomes can be internalized through phagocytosis by both phagocytic and non-phagocytic cells, and this mechanism requires the presence of specific receptors on the plasma membrane of the recipient cell. As described by Feng et al., phagocytosis appears to be dependent on actin cytoskeleton because the actin polimerization is a key mechanism for phagosome formation. Moreover, phosphatidylinositol 3-kinase (PI3K) seems to facilitate exosome membrane insertion into the phagosome, and the dynamin2 is an important regulator of phagocytosis. Dynamin2 is a GTPase also required for clathrin-mediated endocytosis, a receptor-mediated process by which exosomes internalization occurs [26]. Indeed, dynamin2 facilitates the process through which clathrin-coated vesicles induce the invagination of the membrane, which collapses into a vesicular bud [27]. Endocytosis can also depend on caveolae, a sub-domain of glycolipid rafts or involved lipid rafts of the plasma membrane, rich in protein receptors and sphingolipids. Clathrin-independent endocytosis appears to require the presence of cholesterol found in lipid rafts. While, during macropinocytosis, plasma membrane protrusions driven by actin filaments form an invagination which endocytoses extracellular fluid and small particles in a non-specific manner, the delivery of exosome content to the acceptor cell, through fusion with the plasma membrane, is the final step of EV uptake [28] (Figure 1). As described in a recent study, acidic pH of the endosome facilitates the fusion of the exosomes with another membrane, rather than the neutral pH of the plasma membrane [29].

Moreover, it has been reported that exosomes can transfer information to recipient cells without delivery of their content, acting directly at the cell surface. For example, cellular responses induced by soluble and juxtacrine signalings do not require internalization but depend on the proteolytic cleavage of exosomal ligands or on the position of ligands and receptors on the exosome surface and in target cells, respectively [30]. Alternatively, exosomes can also be internalized and targeted to the lysosomes for degradation or recycling and released in the extracellular space.

## 3. Role of Exosomes in Breast Cancer Metastasis

Growing evidence has highlighted the contribution of exosome-mediated signaling in all the aspects of breast cancer metastatic dissemination. Metastasis is an extremely intricate biological process during which primary cancer cells must: (i) invade adjacent tissues; (ii) enter the blood or lymphatic vessels; (iii) colonize distant target organs where they can proliferate and form clinically detectable neoplastic growths. The findings outlined by the current literature in the field are summarized in Table 1. 

The initial event of the metastatic process involves the local invasion of primary tumor cells into the surrounding tissues through the remodeling of the extracellular matrix (ECM) and the acquisition of migratory capacity [64]. During these events, primary epithelial tumor cells undergo multiple biochemical and morphological changes resulting in loss of the epithelial characteristics (e.g., polarity and cell–cell junctions) to achieve a mesenchymal phenotype. This process, known as epithelial–mesenchymal-transition (EMT), that enables the physical dissemination of cancer cells, is influenced by different EMT-inducing signals coming either from the tumor cell itself but also from the surrounding stromal cells in the complex tumor microenvironment (TME).

### 3.1. ECM Remodeling and EMT 

Several proteins, signaling molecules and miRNAs, regulating cytoskeleton remodeling, cell motility and invasion capability, have been identified in the cargos of exosome-like vesicles released from breast cancer cell lines [65,66]. An interesting comparative analysis on the proteomic profile of exosomes isolated from several mouse breast cancer cell lines, revealed that metastatic cell-derived exosomes contain protein involved in the migration, invasion and angiogenesis pathways, including ceruloplasmin and metadherin, which could be potentially involved in directing the primary tumor cells to specific metastatic sites [67]. The proteomic profile of exosomes secreted by MDA-MB-231, the cell line derived from the malignant pleural effusion of a patient with invasive breast cancer, revealed an enrichment of the enzymes responsible of degrading the extracellular matrix, the matrix-metalloproteinases (MMPs), which might be linked to the enhanced metastatic property of cells [65]. In line with this observation, it has been reported that the transfer of the EVs from highly metastatic MDA-MB-231 breast cancer cells to non-malignant breast epithelial cells MCF-10A, induced an increased secretion of MMP-2 and MMP-9 from the recipient cells together with an EMT phenotype characterized by a reduced expression of the epithelial marker E-cadherin and an increased expression of several mesenchymal markers (e.g., SNAIL, TWIST, vimentin and N-cadherin) [31]. Moreover, it has been demonstrated that EVs of MDA-MB-231 cells contain Caveolin-1, a membrane protein promoting the migration and invasion of cancer cells, along with other adhesion-related proteins (Cyr61, tenascin and S100A9), that can enhance the migration and invasion capability of recipient cells [32]. In addition to protein content, exosomal miRNA profiling has been reported for many breast cancer cell lines and breast cancer patients [68,69], and an EV miRNA database of miRNA profiling has been created for public searching [70]. For instance, specific exosomal miRNA signatures have been found in triple-negative and HER2-positive breast cancer patients, and have been associated with the clinicopathological parameters and aggressiveness of the disease [69]. An in vitro study, using exosomal miRNAs from MDA-MB-231 demonstrated that the internalization of miRNAs in non-metastatic MCF-7 cells resulted in an increased ability of MCF-7 cells to grow in an anchorage-independent manner, and to acquire metastatic behavior [33]. Singh et al. revealed that the transfer of exosomes from MDA-MB-231 cells promotes invasion in non-malignant HMLE cells mediated by the inhibition of the tumor suppressor miR-10b homeobox D10 (HOXD10) and Krüppel-like factor 4 (KLF4) protein expression [34]. Similarly, exosomal miR-1246 from breast cancer cells can suppress the expression of its target gene, Cyclin-G2 (CCNG2), enhancing the viability and migration in normal epithelial cells [35]. An additional miRNA such as miR-155, an oncomiR involved in Transforming Growth Factor beta (TGF-β)-induced EMT, has been proposed as a component of exosomal cargo able to promote breast cancer progression. Indeed, exosomes secreted from breast cancer stem cells (BCSCs) were enriched with miR-155, and the transfer of this miRNA induced a gain of the EMT process in chemo-sensitive breast cancer recipient cells [36]. In addition, EVs released by HER2 breast cancer cells resistant to trastuzumab-dependent cell cytotoxicity display an increased level of the immunosuppressive molecules TGF-β1 and PD-L1 (programmed death-ligand 1) and are able to transfer a resistant phenotype to drug-sensitive cells [71]. In line with this evidence, Ciravolo et al. demonstrated that exosomes released by the HER2-overexpressing breast cancer cell lines bound to trastuzumab inhibit its activity [72]. MDA-MB-231 breast cancer cells ectopically expressing miR-9 secreted exosomes containing this specific miRNA that can be taken up by recipient normal fibroblasts resulting in their enhanced motility. In turn, miR-9 secreted from fibroblasts can sustain the motility of breast cancer cells, thus establishing a positive feedback loop able to increase tumor progression [37]. From the above reported findings, it emerges how exosomes might work through both homotypic transfer between cancer cells as well as heterotopic transfer between cancer and non-malignant cells. In this latter concern, exosomes may act as intriguing components of the TME communication signaling. Indeed, it has been demonstrated that exosomes derived from cancer associated fibroblasts (CAFs) promote protrusive activity and motility in recipient breast cancer cells mobilizing autocrine Wnt-planar cell polarity (Wnt-PCP) signaling [38]. Recently, Wang and coworkers reported that CAF-derived exosomes carrying miR-181d-5p function as promoters in the EMT, invasion and migration of MCF-7 breast cancer cells, reducing the expression of Caudal-related homeobox 2 (CDX2) and homeobox A5 (HOXA5) [39]. Particularly in TME, breast cancer cells communicate with infiltrating macrophages to stimulate a tumor-promoting macrophage phenotype, and in turn, activated macrophages (named tumor associated macrophages-TAMs) can influence epithelial cancer cells mainly by secreting several soluble factors. Additionally, it has been reported that macrophages secrete EVs including exosomes able to shape breast cancer behavior. For instance, interleukine (IL)-4-activated macrophages regulated the invasiveness of MDA-MB-231 and SKBR3 breast cancer cells through the exosome-mediated delivery of oncogenic miR-223 targeting the β-catenin pathway [41]. On the other hand, breast cancer cell-secreted EVs can induce infiltrating macrophages to release Wnt 5a-positive exosomes which then induce invasion in MCF-7 cells, creating a vicious circle [40]. The activation of Wnt signaling that increases migration capability in breast cancer cells was also induced by the human adipose-derived mesenchymal stem cell (MSC) exosomes [42]. Breast cancer-derived exosomes can promote a myofibroblast-like phenotype in MSCs in vitro, through the activation of the TGF-β pathway, leading to the secretion of tumor-promoting factors such as stromal cell derived factor-1 (SDF-1), vascular endothelial growth factor (VEGF), chemokine (C-C motif) ligand 5 (CCL5) and TGF-β [43].

Another interesting issue explored by authors was the impact of circulating exosomes derived from healthy subjects on tumor epithelial cell properties. Studies carried out using exosomes derived from the plasma of healthy female donors demonstrated that these EVs stimulate the adhesive, motile and invasive properties of breast cancer cells in vitro, as well as their metastatic dissemination in the Zebrafish model, via the focal adhesion kinase (FAK) signaling pathway activation [44]. In mice, serum-derived exosomes from highly metastatic 4THM breast cancer tumors were shown to transfer increased metastatic capacity to poorly metastatic EMT6 tumor bearers [45].

### 3.2. Role of Exosomes in Angiogenesis, Intravasation and Immunomodulation

To allow the metastatic process tumor cells, after primary growth, they should be able to invade the basement membrane and enter the circulation avoiding apoptotic signals and host immune responses. Primarily, tumor neoangiogenesis occurs to provide breast cancer cells with more nutrients and oxygen, and to gain access to the route for the metastatic dissemination. Next, cancer cells invade the basement membrane and cross the endothelial barrier passing through endothelial cell junctions resulting in the intravasation into blood or lymphatic vessels. Finally, the limiting step of the successful metastatic process is represented by the survival in the bloodstream where circulating tumor cells (CTCs) are exposed to hemodynamic shear forces, innate immunity response and oxidative stress.

#### 3.2.1. Angiogenesis

It is well established that the crosstalk between breast cancer cells and endothelial cells, mediated by secretion of growth factors, cytokines and induction of hypoxia may facilitate angiogenesis process. Many findings also support the idea that exosomes secreted by cancer cells could transport angiogenic cargo promoting new vascular network formation through the activation of endothelial cells. In vitro and in vivo experimental approaches revealed that exosomal Annexin II, a Ca^2+^-dependent phospholipid-binding protein associated with the plasma membrane, promotes angiogenesis in a tPA-dependent manner in breast cancer cells [46]. The expression of exosomal-annexin A2 was found significantly higher in sera of breast cancer patients compared to non-cancer subjects, particularly in African-American women with Triple-Negative Breast Cancer (TNBC), and was shown to be associated with poorer clinic-pathological features of the breast cancer patients. In addition, an in vivo matrigel plug assay revealed that exosomal-annexin A2 from TNBCs promoted angiogenesis [47]. Moreover, it has been reported that neutral sphyngomyelinase 2 (nSMase2) regulates exosomal miRNA secretion in metastatic breast cancer cells. Exosomes released from these cells are enriched in a set of angiogenic miRNAs such as miR-210 that is transferred to endothelial cells, where it enhances the capillary formation and migration capability [48]. It is well known that intratumoral hypoxia can drive breast cancer neoangiogenesis [73], and now it has also been associated with an enhancement of exosome release from breast cancer cells in a HIF-1α-dependent manner [22]. Exosomes released in hypoxic conditions contain increased amounts of miR-210 [22] that can promote endothelial cell tube formation [74]. On the other hand, it has been previously reported that mesenchymal stem cells (MSC)-derived exosomes negatively modulate angiogenesis in breast cancer cells by down-regulating vascular endothelial growth factor (VEGF) expression in vitro and in vivo. This effect appears partially due to the transfer of exosomal miR-16 to mammary tumor cells [49]. In line with these observations, other authors reported that MSC-derived exosomes were enriched in miR-100 and were able to suppress angiogenesis in vitro, down-regulating VEGF expression through the modulation of the mammalian target of rapamycin (mTOR)/hypoxia-inducible factor 1-alpha (HIF-1α)/VEGF signaling in breast cancer cells [50]. These latter findings highlight a multifaceted role of exosomes in mediating cancer-to-stromal cell communication within TME, suggesting that many aspects in this intricate connection should be further unravelled.

#### 3.2.2. Intravasation

The intravasation process requires the destruction of basement membrane integrity, by perturbing the endothelial cell barrier. The stability of endothelial junctions is maintained by the expression of the membrane-associated adhesion molecules such as claudin-5, occludin, vascular endothelial cadherin (VE-cadherin) and zonula occludens 1 (ZO-1) [75]. Recently, Di Modica et al. demonstrated that exosomes released in vitro from TNBC cells contain miR-939 and negatively regulate VE-cadherin expression in the endothelial cells, HUVECs, leading to an increase in cell monolayer permeability [51]. Interestingly, aspartate β-hydroxylase (ASPH) induces MDA-MB-231 breast cancer cells to secrete exosomes with pro-invasive/pro-metastatic cargo comprising active Notch receptors, ligands, regulators and MMPs. These exosomes, in turn, were able to enhance transendothelial migration and invasion through the basement membrane of breast cancer cells, thus supporting the intravasation process [52].

#### 3.2.3. Immunomodulation and Survival in the Circulation

Evasion of immune surveillance is a crucial step to gain metastatic outgrowth. Many findings revealed that exosomes, using multiple mechanisms, can help breast cancer cells to exert immunomodulatory activities. Exosomes released by murine mammary carcinoma cells TS/A or 4T1 induced cancer growth in a mouse model, inhibiting natural killer (NK) cell cytotoxic activity ex vivo and in vivo [53]. Additionally, murine C57BL/6 EO771 cell-derived exosomes suppress proliferation of both CD8 and CD4 T-cells by inducing apoptosis, and also reduce the cytotoxic activity of NK cells against tumor cells in vitro [54]. Moreover, exosomes derived from two different breast cancer cell lines (MDA-MB-231 and BT-474) in a hypoxic condition exert a potent immunosuppression activity, negatively modulating T-cell proliferation through TGF-β [55]. Recently, an elegant work of Xing and coworkers demonstrated that breast cancer cells reproducing loss of the X-inactive-specific transcript (XIST), MCF7-shXIST, released exosomal miR-503 that promotes the M1 to M2 conversion of microglia through STAT3 and NFκB pathways, inducing the suppression of T-cell proliferation [56]. Indeed, the immunomodulation of macrophage activity represents an important mechanism used by breast cancer cells to promote metastasis. In this contest, it has been reported that breast cancer-secreted exosomes stimulate NF-κB activation in macrophages resulting in the secretion of pro-inflammatory cytokines such as IL-6, tumor necrosis factor alpha (TNFα), Granulocyte colony-stimulating factor (GCSF) and CCL2. This requires the activation of the Toll-like receptor 2 (TLR2) in macrophages and is influenced by the presence of palmitoylated protein ligands on exosome surfaces [57]. Interestingly, exosomes isolated from tumor cells of a mouse model of breast cancer exhibited immune suppression activity inducing bone marrow derived CD11b^+^Gr-1^+^ cells to differentiate in myeloid-derived suppressor cells (MDSCs), known to promote tumor progression, via prostaglandin E2 (PGE2) and TGF-β pathways [58]. Moreover, CTCs, to protect themselves in the circulatory system, can induce platelet aggregation that, in addition to avoiding the immune system surveillance, also facilitates extravasation and arrests at distant target organs. EVs from the highly invasive MDA-MB-231 breast cancer cells have been demonstrated to induce platelet P-selectin exposure and platelet aggregation in a tissue factor-dependent manner [76].

### 3.3. Role of Exosomes in the Pre-metastatic Niche of Distant Sites (Bone, Lung, Liver and Brain) 

As final step of the metastatic process, the CTCs, that have survived blood stresses, anoikis, and host immune response in the circulation, should reach the distant target organs where they must reacquire the epithelial phenotype and thus epithelial cell-to-cell junctions going through a reversal process named mesenchymal–epithelial transition (MET). The EMT/MET transition implies multiple changes in gene expression, mainly regulated by the EMT-associated transcription factors, and is accompanied by a dynamic transdifferentiation process that occurs in different metastatic microenvironments. Several reports suggest that exosomes actively drive organotropic metastasis of cancer cells by altering the metabolism of non-transformed cells and/or educating the cells of the hosting tumor microenvironment (such as endothelial progenitor cells, mesenchymal cells and bone marrow-derived cells), thus establishing the permissive premetastatic niche [62,63,77].

#### 3.3.1. Extravasation

To exit from the circulatory system, a mechanism known as extravasation, cancer cells adhere to vascular endothelium in a distant metastatic secondary site and leave the circulation. The preferred sites of colonization of circulating breast cancer cells are in order bone (65%), lung (31%), liver (26%) and brain (8.8%) [78]. Metastasis organotropism cannot be completely explained by the anatomy of the blood and lymphatic vessels networks, but additional mechanisms can influence the vasculature permeability, the adhesion of CTCs to the lining of the vasculature and their extravasation. For instance, it has been demonstrated that metastatic breast cancer cells secrete exosomes containing miR-105 that efficiently destroys the endothelial barrier integrity through targeting the tight junction protein ZO-1, significantly increasing in vivo metastases in the lung and brain [59]. Accordingly, circulating miR-105 was significantly high in tumor-bearing animals compared to tumor-free animals, and more interestingly, miR-105 was found significantly higher in the serum of breast cancer patients who later developed distant metastases than in the serum of those patients who did not [59]. Moreover, metastatic breast cancer-derived EVs contained miR-181c that promotes the blood–brain barrier (BBB) destruction via the downregulation of the actin regulator 3-phosphoinositide-dependent protein kinase-1 (PDPK1), resulting in increased brain metastasis in a mouse model [60]. More recently, it has been reported that small EVs from MDA-MB-231 cells are able to breach the BBB by increasing the efficiency of brain endothelial cell transcellular transport [61]. 

#### 3.3.2. Metastatic Colonization and Growth 

**Figure 2:**
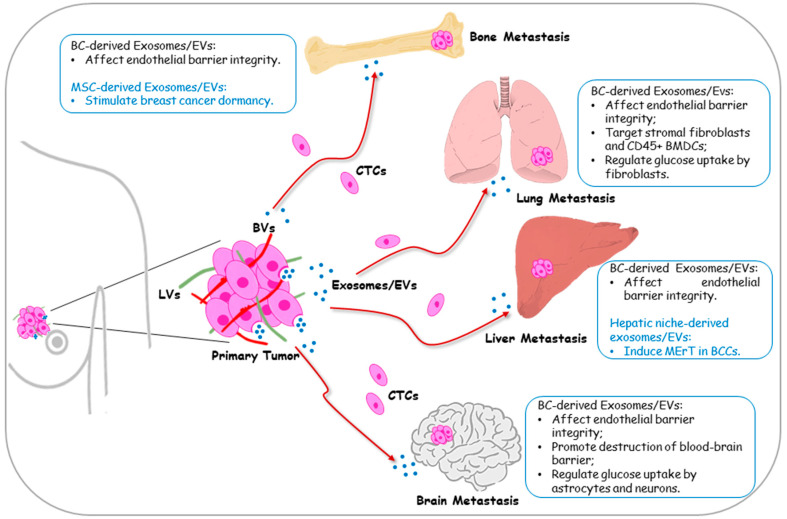
Schematic representation of the effects of exosome/extracellular vesicles (EVs) released by tumor cells and stromal cells in the establishment of metastatic outgrowth in breast cancer. Primary breast cancer-derived exosomes drive organotropic metastasis of circulating tumor cells (CTCs) enabling their survival into blood (BVs) or lymphatic vessels (LVs) and establishing the permissive premetastatic niche at the distant sites: bone, lung, liver, and brain. The main mechanisms involved are summarized in the boxes near each metastatic site.

Lastly, in the metastatic cascade, CTCs that reached the target organ might proliferate and become clinically relevant tumor mass. To accomplish the development of metastatic tumors, CTCs (“seed”) can find a receptive microenvironment in distant target organs (“soil”). Indeed, it is now clear that the specificity of the pre-metastatic niche microenvironment is critical in the fate of CTC outgrowth at the distant site, and several reports indicate a role for tumor-derived exosomes in preparing the “soil” for future metastatic seeding. Exosome labelling experiments using CD63-GFP expressing breast cancer cells in orthotopic nude mice showed that tumor-derived exosomes can be found circulating in the blood of mice as well in lung metastases, where they target stromal cells such as fibroblasts [79]. Optical imaging was also used to follow the distribution of exogenously administered fluorescently labeled exosomes derived from highly metastatic murine breast cancer cells in mice. Labeled exosomes were distributed predominantly to the lung, where they were taken up by CD45+ bone marrow derived cells, and created a favourable immune suppressive TME sustaining metastatic colonization of the site [54]. A reprogrammed metabolism in the tumor microenvironment is a well-known hallmark of cancer. Breast cancer exosomal miR-122 regulates glucose uptake by non-tumor cells (e.g., lung fibroblasts, brain astrocytes and neurons) in the pre-metastatic niche thus increasing nutrient availability to metastatic seeding cells, thus facilitating disease progression [62]. Feng et al. reported that breast cancer cell-secreted exosomes induce lung fibroblasts proliferation and migration, and through high-throughput sequencing analysis of exosomal content, they identified long non-coding RNAs involved in the establishment of the pulmonary pre-metastatic niche [63]. A role for exosomes was proposed in tumor “self-seeding”, a process by which cancer cells become able to reinfiltrate the original tumor. Indeed, it has been demonstrated that exosomes derived from a subpopulation of breast cancer lung metastasis, the MDA231-LM2, potentiate the growth of MDA-MB-231 in xenograft models [80]. In an elegant work, Hoshimo et al. proved, in different mouse models, that the organ specificity of exosome biodistribution corresponds to the organotropic distribution of the cell line of origin. The authors proposed that unique exosomal integrins direct organ-specific colonization initiating pre-metastatic niche formation. Particularly, they found the highest levels of integrin β_4_ in exosomes isolated from breast cancer patients that develop lung metastasis [77]. Moreover, it has been demonstrated that the role of hypoxia-induced exosomes in stimulating breast cancer invasion and lung metastasis is by a mechanism involving RAB22A expression, since hypoxia mediated ECM invasion and lung colonization is lost after RAB22A knockdown [81]. In addition, pre-metastatic niche-derived exosomes can influence tumor outgrowth or dormancy of the CTCs. Exosomes from the hepatic niche negatively affected MDA-MB-231 and MDA-MB-468 breast cancer cell proliferation and invasion, and induced Mesenchymal to Epithelial reverting Transition (MErT), increasing E-cadherin and ZO-1 protein expression levels in both breast cancer lines [82]. Recently, it has also been reported that the ability of breast cancer cells to spontaneously undergo EMT/MET transition was inhibited by exosomes released by MDA-MB-231 basal cells [83]. While it is clear that breast cancer can metastasise easily into the bones, the role of breast cancer-derived exosomes in governing this process has not been clarified yet. On the other hand, it has been reported that breast cancer cells prime MSCs to secrete exosomes containing mir-222/223, which in turn stimulate early breast cancer dormancy in bone marrow [84]. The main mechanisms by which exosome/EVs may influence metastatic organotropism are summarized in Figure 2.

## 4. Conclusions

Metastatic breast cancer is still considered an incurable disease that needs new early diagnostic tools and more therapeutic options to become a less deadly malignancy. Thus, tremendous efforts have been focused on the better understanding of the mechanisms by which breast cancer cells escape the primary tumor, survive in the circulation, overcome immune surveillance and initiate outgrowth at secondary organs. A large amount of literature has reported that the dynamic interaction among the different cell types in the local as well as in the distant tumor microenvironment regulates various stages of breast cancer metastasis, and exosomes have been largely demonstrated to mediate many of the involved processes. Indeed, breast cancer-derived exosomes directly participate in cancer cell plasticity by increasing tumor cell motility and ECM degradation. Moreover, the discussed findings support the idea that tumor-derived exosomes may contribute to metastatic organotropism, creating a permissive niche in specific metastatic secondary sites. Although, based on the current knowledge, mainly obtained from in vitro studies, exosome activity is relevant in breast cancer metastasis, several questions arise: How and when do exosomes reach the premetastatic niche during the disease progression? How do cancer cells acquire the ability to release specific exosomal cargo that in turn sustains breast cancer plasticity and tumor metastatic spread? Can the detection of cancer-derived exosomes be used as a marker for the early diagnosis of metastasis? May exosomes from other tissues affect the metastatic process (e.g., exosomes from fat mass)? To answer these and other questions, further studies should be carried out using genetically engineered animal models that allow the following of circulating exosome routes without external administration of vesicles that could represent an artefact. In addition, the evaluation and characterization of circulating exosomes in breast cancer patients at different stages of disease should be addressed. Overall, the biology of exosomes is still an open discussion to be covered and represents an attractive research area to be explored for novel opportunities in prevention, diagnosis and therapeutic approaches in breast cancer.

## 5. Review Criteria

A search for original articles published in the last two decades was performed in PubMed. We used the following search terms: “exosome biogenesis”, “exosome uptake”, “exosomes and breast cancer”, “exosomes and breast cancer and metastasis”, “exosomes and breast cancer and EMT”, “exosomes and breast cancer and angiogenesis”, “exosomes and breast cancer and immunomodulation”, “exosomes and breast cancer and intravasation”, “exosomes and breast cancer and extravasation”, “exosomes and breast cancer pre-metastatic niche”, “exosomes and breast cancer and organotropism”. Furthermore, we selected additional relevant original articles from the reference lists of the used papers. All the articles selected were English-language full-text papers.

## Figures and Tables

**Table 1:** Exosome-mediated mechanisms sustaining metastatic process in breast cancer.

Source of Exosomes /EVs	Recipient Cells	Molecules and Signalings	Biological Effects	Ref.
MDA-MB-231 cells	MCF-10A cells	MMP-2, MMP-9	Increased EMT phenotype	[31]
MDA-MB-231 cells	BCCs (CAV negative)	Caveolin-1, Cyr61, Tenascin, S100A9	Increased migration and invasion	[32]
MDA-MB-231 cells	MCF-7 cells	miRNAs	Increased metastatic potential	[33]
MDA-MB-231 cells	HMLE cells	miR-10b	Increased invasion	[34]
MDA-MB-231 cells	HMLE cells	miR-1246	Increased viability and migration	[35]
BCSCs	MCF-7 cells	miR-155	Increased EMT phenotype	[36]
MDA-MB-231 cells	Normal fibroblasts	miR-9	Increased motility	[37]
CAFs	MDA-MB-231 cells	Wnt-PCP	Increased protrusive activity and motility	[38]
CAFs	MCF-7 cells	miR-181d-5p	Increased EMT phenotype	[39]
Macrophages	MCF-7 cells	Wnt 5a	Increased invasion	[40]
Macrophages	MDA-MB-231 cells, SKBR3 cells	miR-223	Increased invasion	[41]
Adipose MSCs	MCF-7	Wnt signalling	Increased migration	[42]
MCF-7, MDA-MB-231 cells	MSCs	TGF-β pathway	Increased myofibroblast-like phenotype	[43]
Plasma of healthy female	MCF-7, MDA-MB-231 cells	FAK signalling	Increased migration and invasion	[44]
Serum of mice bearing 4THM tumors	Poorly metastatic EMT6 cells	miR-155, miR-205	Increased metastatic potential	[45]
MCF-10CA1a cells	HUVECs	Annexin II	Increased angiogenesis	[46]
BC patients serum	Endothelial cells	Annexin A2	Increased angiogenesis	[47]
Metastatic BCCs	Endothelial cells	miR-210	Increased capillary formation and migration capability	[22,48]
MSCs	4T1 cells	miR-16	Decreased VEGF expression	[49]
MSCs	MCF-7, MDA-MB-231 cells	miR-100	Decreased VEGF expression	[50]
TNBCs	HUVECs	miR-939	Decreased VE-cadherin expression	[51]
MDA-MB-231 cells	BCCs	Notch signalling, MMPs	Increased transendothelial migration and invasion	[52]
TS/A cells, 4T1 cells	NK		Decreased cell cytotoxic activity	[53]
EO771 cells	CD8, CD4 T cells and NK		Increased Apoptosis, and decreased proliferation and cell cytotoxic activity	[54]
MDA-MB-231, BT-474 cells	T-cells	TGF-β	Decreased proliferation	[55]
MCF7-shXIST cells	Macrophages	miR-503	Increased M1 to M2 polarization	[56]
MCF-7, MDA-MB-231 cells	Macrophages	palmitoylated protein ligands	Increased pro-inflammatory activity	[57]
TS/A, 4T-1 cells	CD11b^+^Gr-1^+^ cells	PGE2 and TGF-β pathways	Increased differentiation in MDSCs	[58]
Metastatic BCCs	Endothelial cells	miR-105	Increased metastases in the lung and brain	[59]
Metastatic BCCs	BBB cells	miR-181c	Increased metastasis in brain	[60,61]
MDA-MB-231 cells	Lung fibroblasts, brain astrocytes, neurons	miR-122	Decreased glucose uptake	[62]
MDA-MB-231 cells	Lung fibroblasts	lncRNAs	Increased metastasis in lung	[63]

EVs, extracellular vesicles; BCCs, Breast Cancer Cells; HMLE, Human Mammary Epithelial cells; BCSCs, Breast Cancer Stem Cells; CAFs, Cancer Associated Fibroblasts; MSCs, Mesenchymal Stem Cells; TNBCs, Triple Negative Breast Cancers; NK, Natural Killer; PGE2, Prostaglandin E2; TGF-β, Transforming Growth Factor beta; FAK, Focal Adhesion Kinase; MMP-2, Matrix Metalloproteinase-2; MMP-9, Matrix Metalloproteinase-9; M1, tumor suppressive macrophages; M2, tumor promoting macrophages.
